# Japanese Nationwide Questionnaire Survey on the Treatment and Management of Subarachnoid Hemorrhage Due to Ruptured Cerebral Aneurysm

**DOI:** 10.3390/jcm14124107

**Published:** 2025-06-10

**Authors:** Toshikazu Hidaka, Junichiro Ochiai, Yusuke Inoue, Yuichiro Kawamoto, Nobutaka Horie, Yusuke Nishikawa, Mitsuhito Mase, Motohiro Morioka, Jun C. Takahashi, Hiroaki Shimizu, Fusao Ikawa

**Affiliations:** 1 Department of Neurosurgery, Shimane Prefectural Hospital, Izumo 693-0068, Japan; thidaka55@yahoo.co.jp (T.H.);; 2 Department of Neurosurgery, Graduate School of Biomedical and Health Sciences, Hiroshima University, Hiroshima 734-8553, Japan; 3 Department of Neurosurgery, Nagoya City University Graduate School of Medical Science, Nagoya 467-8601, Japanmitmase@med.nagoya-cu.ac.jp (M.M.); 4 Department of Neurosurgery, Kurume University School of Medicine, Kurume 830-0011, Japan; 5 Department of Neurosurgery, Kindai University Faculty of Medicine, Osaka-Sayama 589-8511, Japan; 6 Akita Prefectural Hospital Organization, Akita 010-0874, Japan

**Keywords:** cerebral vasospasm, clazosentan, delayed cerebral ischemia, subarachnoid hemorrhage, survey

## Abstract

**Background**: Since clazosentan was approved for insurance coverage in Japan, the postoperative management of delayed cerebral ischemia (DCI) after subarachnoid hemorrhage (SAH) has changed as each facility gains experience. Here, we investigate the prevention, treatment, and management of DCI after SAH throughout Japan in 2023. **Methods**: In 2024, we conducted an anonymous questionnaire survey—emailed to certified neurosurgeons in hospitals across Japan—regarding management for preventing DCI after aneurysmal SAH. Of them, 78 hospitals responded and were included in this study. These results were compared with the findings of a survey conducted prior to the approval of clazosentan in Japan (2022). **Results**: The proportion of institutions with a standardized protocol for DCI after aneurysmal SAH at a level of ≥50% was 93.0%. For both craniotomy and endovascular surgery, clazosentan was used most frequently, followed by cilostazol, fasudil, and statins. The most common drug for both direct and endovascular procedures was clazosentan. The predominant reason for discontinuing clazosentan was respiratory complications—such as pulmonary edema—followed by cardiac complications. However, 62.1% of facilities felt that the number of cases wherein clazosentan was discontinued was deceasing. While 77.5% of respondents felt that clazosentan was effective for preventing DCI after aneurysmal SAH, only 49.3% felt that it improved outcomes. **Conclusions**: Since its approval, clazosentan has been the most common treatment for DCI prevention after aneurysmal SAH. The impression of the effectiveness in preventing DCI and the outcomes of clazosentan have been mixed. As data accumulate, clazosentan use and management protocols will be refined and developed.

## 1. Introduction

Cerebral vasospasm (CVS) and delayed cerebral ischemia (DCI) remain the major causes of disability and death after subarachnoid hemorrhage (SAH) [1,2]. Despite advances in neurocritical care, they continue to be regarded as significant challenges in the management of patients with SAH. To date, various management methods—including the use of medications such as nimodipine and stains—have been used to treat DCI globally. The American Heart Association/American Stroke Association revised their guidelines for the management of patients with SAH in 2023, recommending the use of nimodipine [3]. The Japanese guidelines for the management of SAH recommended various medications for DCI, including fasudil, ozagrel, and cilostazol [4]. Clazosentan—a currently available selective endothelin receptor antagonist—was approved for use in 2022 [5,6,7,8,9], dramatically changing treatment in Japan. A survey conducted in Japan in 2021 [10] found that clazosentan was already being used in clinical studies at some hospitals; however, its actual use in Japan is unknown.

In this study, we aimed to elucidate current practices regarding the prevention, treatment, and management of CVS following SAH since the introduction of clazosentan in Japan. We also aimed to compare the results of a survey conducted in 2021 [10] before the introduction of clazosentan, using a questionnaire survey administered to hospitals with neurosurgeons certified by the Japanese Society on Surgery for Cerebral Stroke.

## 2. Materials and Methods

The Institutional Review Board of Shimane Prefectural Central Hospital approved this study (approval no. R24-003). All procedures including human subjects were conducted in accordance with the “Ethical Guidelines for Medical and Health Research Involving Human Subjects (Provisional Translation as of March 2015)” and their later amendment.

In October 2024, we conducted an anonymous questionnaire survey regarding treatment methods for preventing DCI associated with CVS after aneurysmal SAH. The questionnaire was administered to cerebrovascular neurosurgeons certified by the Japanese Society on Surgery for Cerebral Stroke who performed neurosurgical surgery to prevent the re-rupture of ruptured cerebral aneurysms within 72 h of onset between January and December 2023. The list of hospitals and neurosurgeons was obtained from the electronic database published by the Japanese Society on Surgery for Cerebral Stroke and was distributed via email to the registered email addresses provided to the Japanese Society on Surgery for Cerebral Stroke. The number of stroke patients treated at these hospitals is required to be registered annually to the Japanese Society on Surgery for Cerebral Stroke as part of their annual clinical survey. To avoid requiring respondents to extract data directly from patients’ medical records for this study, the questionnaire was designed to be completed solely based on data previously submitted to the annual survey. The survey collected information regarding the location of the institution, the number of treated cases (both craniotomy and endovascular treatments), the types of drugs used, and perioperative management strategies. If a facility had multiple certified cerebrovascular neurosurgeons, one representative of the facility was to answer the questions (Supplementary Materials) by using Google Forms. The responders were instructed to answer in single- or multiple-choice format regarding the management of SAH, and the results were compared with the findings of a survey conducted in 2022 [10], prior to the approval of clazosentan in Japan.

## 3. Results

### 3.1. Institutional Information and Case Numbers: Questions 1–3

A total of 1316 patients from 78 hospitals were ultimately included in this study. Personal information—including names, ages, and genders—were not collected; thus, approval by an institutional review board (IRB) was not required. To minimize the risk of patient identification, only the geographic region of each responding hospital was recorded; the names of the hospitals were not collected. The participating hospitals were distributed across Japan, allowing for nationwide data collection, as follows: Hokkaido (*n* = 3), Tohoku region (*n* = 11), Kanto region (*n* = 30), Chubu region (*n* = 8), Kinki region (*n* = 15), Chugoku-Shikoku region (*n* = 6), and Kyushu region (*n* = 5) (Table 1).

### 3.2. Protocol Availability: Question 4

**Figure 1:**
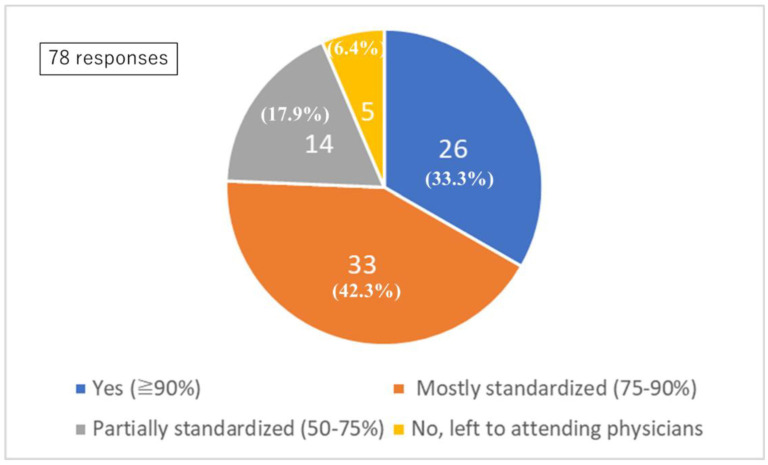
Standardized protocol. Q4: Standardized protocol for postoperative management and vasospasm prevention.

In 33.3% of institutions, more than 90% of the postoperative management and prevention as well as treatment of CVS was determined by standardized protocols, with less than 10% left to the discretion of the attending physician. Furthermore, 42.3% of institutions conducted treatment primarily (75–90%) in accordance with established protocols, allowing 10–25% of treatment to be made at the physician’s discretion. In 17.9% of institutions, treatment was guided by protocols in more than half (50–75%) of treatments, with 25–50% left to the attending physicians’ judgment. In 6.4% of institutions, management was entirely at the discretion of the attending physician (Figure 1).

The proportion between protocol-based management and physician-directed management was judged by each institution.

### 3.3. Pharmacological Treatment: Questions 5–11

Among the 71 patients who underwent craniotomy, the drugs that were predominantly used (>80% of cases) during the first 14 days after onset included clazosentan in 47 cases (66.2%), cilostazol in 34 cases (47.9%), fasudil hydrochloride in 34 cases (47.9%), statins in 28 cases (39.4%), nicardipine (for purposes other than blood pressure control) in 15 cases (21.1%), edaravone in 13 cases (18.3%), and eicosapentaenoic acid (EPA) preparations in 10 cases (14.1%).

Among the 68 patients who underwent endovascular surgery, the drugs predominantly used (>80% of cases) during the first 14 days after onset included clazosentan in 42 cases (61.8%), cilostazol in 40 cases (58.8%), fasudil hydrochloride in 30 cases (44.1%), statins in 26 cases (38.2%), nicardipine (for purposes other than blood pressure control) in 12 cases (17.6%), edaravone in 10 cases (14.7%), and EPA preparations in 7 cases (10.3%).

Among the 62 patients who underwent craniotomy, the drugs that were frequently used (50–80% of cases) during the first 14 days after onset included clazosentan in 30 cases (48.4%), cilostazol in 22 cases (35.5%), fasudil hydrochloride in 32 cases (51.6%), statins in 15 cases (24.2%), nicardipine (for purposes other than blood pressure control) in 7 cases (11.3%), edaravone in 7 cases (11.3%), and EPA preparations in 17 cases (11.3%).

**Figure 2:**
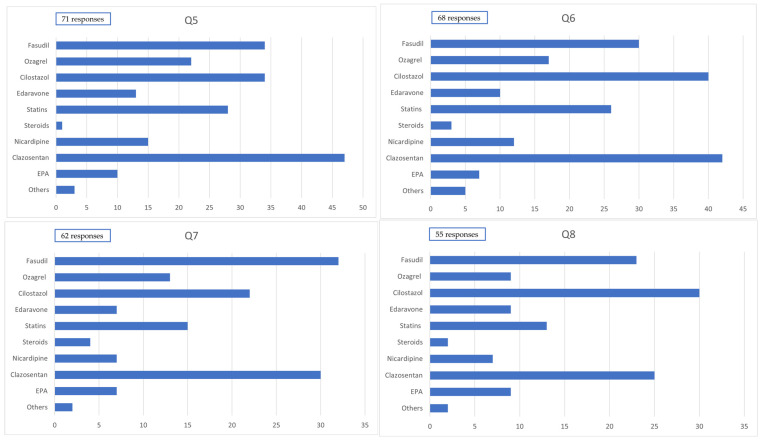
Administration of medication. Q5: Medication administered to ≥80% of cases within 14 days postcraniotomy. Q6: Medication administered to ≥80% of cases within 14 days postendovascular treatment. Q7: Medication administered to 50–80% of cases within 14 days postcraniotomy. Q8: Medication administered to 50–80% of cases within 14 days postendovascular treatment.

Among the 55 patients who underwent endovascular surgery, the drugs that were frequently used (50–80% of cases) during the first 14 days after onset included clazosentan in 25 cases (45.5%), cilostazol in 30 cases (54.5%), fasudil hydrochloride in 23 cases (41.8%), statins in 13 cases (23.8%), nicardipine (for purposes other than blood pressure control) in 7 cases (12.7%), edaravone in 9 cases (16.4%), and EPA preparations in 9 cases (16.4%) (Figure 2).

In a 2021 survey reported by Nishikawa et al. [10], fasudil was used in all 150 cases (100%), followed by cilostazol in 86 cases (57%), ozagrel sodium in 83 cases (55.3%), statins in 49 cases (32.5%), nicardipine in 37 cases (24.5%), edaravone in 25 cases (16.6%), and EPA preparations in 16 cases (10.6%). Clazosentan, which had not yet been approved in Japan at the time, was used in only 7 cases (4.6%) as part of clinical research.

Following the approval of clazosentan in Japan, its use has increased, often replacing fasudil; accordingly, the usage rates of other drugs have generally declined. The proportion of institutions that reported differences in the medication administered to severe (World Federation of Neurosurgical Societies [WFNS] grades IV and V) and mild cases was 11.5% (9 of 78).

Among patients with severe conditions who underwent craniotomy, the predominantly used drugs (>80% of cases, reported across nine responses) were clazosentan in 2 cases (22.2%), cilostazol in 5 cases (55.5%), fasudil hydrochloride in 6 cases (66.7%), statins in 6 cases (66.7%), nicardipine (for purposes other than blood pressure control) in 4 cases (44.4%), edaravone in 2 cases (22.2%), and EPA preparations in 3 cases (33.3%).

**Figure 3:**
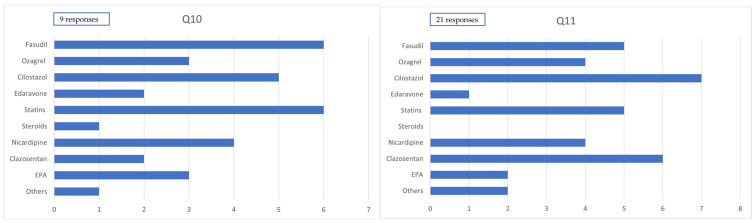
Administration of medication in severe cases. Q10. Medication administered to ≥80% of severe cases within 14 days postcraniotomy. Q11. Medication administered to 50–80% of severe cases within 14 days postendovascular treatment.

Among patients with severe conditions who underwent endovascular surgery, the predominantly used drugs (>80% of cases, reported across 11 responses) were clazosentan in 6 cases (54.5%), cilostazol in 7 cases (63.6%), fasudil hydrochloride in 5 cases (45.5%), statins in 5 cases (45.5%), nicardipine (for purposes other than blood pressure control) in4 cases (36.4%), edaravone in 1 case (9.1%), and EPA preparations in 2 cases (18.2%) (Figure 3).

### 3.4. Cerebrospinal Fluid (CSF) Drainage Management: Questions 12–14

Among the 78 institutions surveyed, 12 (15.4%) reported performing perfusion therapy (e.g., drug administration via CSF drainage tubes for purposes other than CSF removal). In cases involving craniotomy, CSF drainage methods were reported in 72 responses: lumbar drainage alone in 21 cases (29.2%), cisternal drainage alone in 20 cases (27.8%), combined lumbar and cisternal drainage in 6 cases (8.3%), combined lumbar and ventricular drainage in 5 cases (6.9%), combined cisternal and ventricular drainage in 8 cases (11.1%), ventricular drainage alone in 13 cases (18.1%), and no routine use of CSF drainage in 12 cases (16.7%).

**Figure 4:**
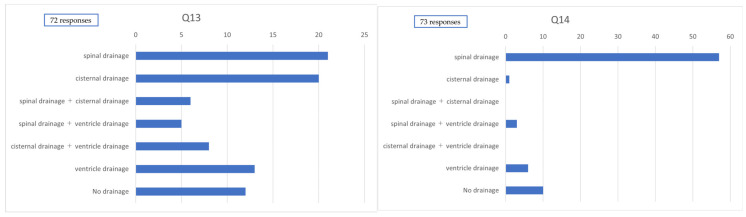
Method of cerebrospinal fluid drainage. Q13. Method of cerebrospinal fluid drainage used postcraniotomy. Q14. Method of cerebrospinal fluid drainage used postendovascular treatment.

In cases involving endovascular surgery, CSF drainage methods were reported in 73 responses, with lumbar drainage alone being the most common (57 cases [78.1%]). Cisternal drainage alone was reported in 1 case (1.4%), and combined lumbar and ventricular drainage in 3 cases (4.1%); combined lumbar and cisternal drainage, as well as combined cisternal and ventricular drainage, were not reported. Ventricular drainage alone was used in 6 cases (8.2%), while 10 cases (13.7%) reported no routine use of CSF drainage (Figure 4).

### 3.5. Measurement of Central Venous Pressure (CVP): Question 15

Water balance is an important factor in the management of CVS. To assess intravascular volume status, some institutions utilize CVP monitoring or inferior vena cava (IVC) diameter measurements. Among the 78 institutions surveyed, 18 reported performing CVP monitoring.

### 3.6. Clazosentan Usage: Questions 16–28

Regarding the concomitant use of clazosentan and fasudil, 10.3% of institutions reported routine coadministration, while 83.3% reported that they do not routinely use both agents together. Additionally, 6.4% of institutions initially used both agents concomitantly, but subsequently discontinued the combination (based on 78 responses). Among the institutions that discontinued coadministration, the primary reasons cited were pulmonary complications in seven cases (77.8%) and cardiac complications in four cases (44.4%; based on nine responses).

Regarding the perceived efficacy of clazosentan for the prevention of CVS, 22 (28.9%), 37 (48.7%), 15 (19.7%), and 2 (2.6%) institutions answered “highly effective”, “effective”, “uncertain”, and “not very effective”, respectively (based on 76 responses). Overall, 77.6% of institutions perceived clazosentan to have a certain level of effectiveness in vasospasm prevention.

**Figure 5:**
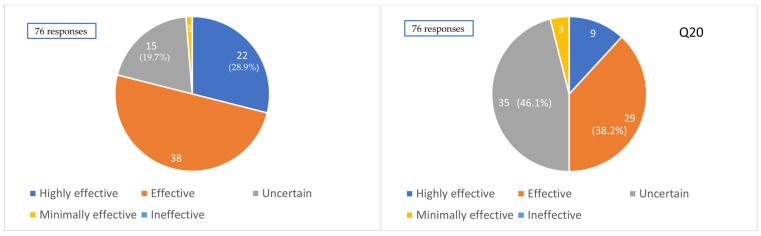
Impression of the efficacy of clazosentan. Q19. Clinical impression of the effectiveness of clazosentan for preventing cerebral vasospasm. Q20. Clinical impression of the effectiveness of clazosentan for improving outcomes.

Conversely, regarding the impact of clazosentan on clinical outcomes, 9 (11.8%), 29 (38.2%), 35 (46.1%), and 3 (3.9%) institutions answered “highly effective”, “effective”, “uncertain”, and “not very effective”, respectively (based on 76 responses). Thus, only 50% of institutions perceived clazosentan as contributing to improved clinical outcomes (Figure 5).

Clazosentan has been associated with adverse effects, including pulmonary edema and heart failure, which in some cases necessitate discontinuation of the drug. The reasons for discontinuation of clazosentan during treatment were as follows: pulmonary complications, such as pulmonary edema, in 30 cases (60%); cardiac complications, such as heart failure, in 16 cases (32%); cerebral edema in 14 cases (28%); and other forms of edema in 4 cases (8%), based on 50 responses.

## 4. Discussion

CVS and DCI remain significant challenges that impact outcomes following SAH. The present study revealed that a wide variety of treatment strategies are employed across different institutions in Japan. While some reports have demonstrated the efficacy of fasudil and statins, a nationwide survey conducted by Nishikawa et al. [10] in 2021 found that only cilostazol was significantly associated with a reduced incidence of DCI. The most effective combination of prophylactic agents identified in that survey was cilostazol, fasudil, and statins.

In 2022, clazosentan—a selective endothelin receptor antagonist—was approved for clinical use in Japan. Since then, its nationwide implementation has contributed to a shift in treatment practices for DCI. In the current study, 33.3% of institutions reported having established clinical protocols covering > 90% of SAH postoperative management items, while 42.3% of institutions reported adherence to standards for approximately 75–90% of these items.

Following both craniotomy and endovascular treatment, the most commonly used agent in principle was clazosentan, followed by cilostazol and fasudil; other agents—including statins, ozagrel sodium, and EPA preparations—were also used. In the analysis of agents used in principle (≥80% of cases) and frequently (50–80% of cases), the use of cilostazol was consistently higher in cases treated with endovascular therapy than craniotomy. This trend is presumed to be due to concerns about thrombotic complications following coil embolization.

In the 2021 nationwide survey, fasudil was reportedly used in principle at all institutions [4]; however, the proportion of institutions that used fasudil in principle declined to 40% in the present study. This suggests that clazosentan has largely replaced fasudil as the primary agent for the management of CVS after SAH.

Among the 78 institutions surveyed, 9 (11.5%) reported altering their pharmacological management based on the severity of a patient’s condition. Among these institutions, fasudil and statins were each used in principle in six institutions (66.7%), and cilostazol in five institutions (55.6%) for patients with severe SAH who underwent craniotomy. In contrast, clazosentan was used in principle in only two institutions (22.2%), suggesting a tendency to withhold clazosentan in severe cases following craniotomy in facilities that adjust medications according to severity. Conversely, in cases managed with endovascular treatment, there was little difference in drug selection between severe and nonsevere cases. This discrepancy may be due to increased concerns about postoperative cerebral edema following craniotomy, which may influence clinicians to avoid clazosentan in such settings.

CSF perfusion therapy involves the intrathecal administration and circulation of pharmacological agents via CSF spaces using combinations of cisternal, ventricular, and lumbar drainage. Although some reports have demonstrated its clinical efficacy [11,12], only 15.4% of institutions in the present study implemented this therapy. It is considered that this approach has not become a standard treatment due to the complexity of its management and concerns about complications, such as infections.

Regarding CSF drainage methods, various approaches were utilized in craniotomy cases, including lumbar, ventricular, and cisternal drainage, or combinations thereof. In contrast, lumbar drainage alone was most commonly employed in endovascularly treated cases. According to the Japanese Guidelines for the Management of Stroke, cisternal drainage is considered appropriate during craniotomy, whereas lumbar or ventricular drainage may be considered in the context of endovascular treatment [4]. Fluid management is critical in the prevention of DCI; however, it remains a challenging task due to the need to account for fluid intake, urinary output, and factors such as fever and insensible perspiration. Additionally, patient-specific variables—such as age and body weight—further complicate accurate assessment.

To evaluate intravascular volume, the CVP and IVC diameter can be measured. In this study, only 23.1% of institutions reported the use of CVP monitoring. While CVP can be easily measured via the placement of a central venous catheter, recent trends favor enteral nutrition—via oral intake or nasogastric tubes—due to the growing recognition of its benefits [13] and concerns over catheter-related infections. As a result, reliance on central venous nutrition has declined, which may account for the decreased use of CVP monitoring.

The combination of clazosentan and fasudil remains controversial [14,15]. In this study, only 10.3% of institutions reported using this combination in standard practice. The high cost associated with dual therapy may be a contributing factor; however, further studies are needed to evaluate the efficacy and cost-effectiveness of this combined approach.

In real-world clinical settings, although 77.6% of institutions perceive clazosentan to be effective in preventing cerebral vasospasm, only 50% believe it contributes to improved clinical outcomes. Its cost-effectiveness also remains to be fully evaluated, warranting further investigation based on future research findings.

Despite the use of clazosentan, various factors were identified to contribute to the development of moderate to severe CVS. Among these, the female sex (76.9%) was the most frequently reported, followed by advanced age (particularly an age ≥ 75 years; 61.5%). Both Xiao et al. [16] and Yang et al. [17] reported that although the incidence of DCI was higher in females, it did not have an impact on clinical outcomes. The potential sex-based differences in the efficacy of clazosentan remain unclear, and further research is warranted to elucidate this aspect.

This study has several limitations: First, detailed information on individual SAH patients—such as severity, imaging findings, and outcomes—was not available; only hospital-level data could be obtained. Second, the study included data from 1316 cases among 78 institutions, which represent a relatively small sample size. Additionally, although data were collected from institutions across Japan, the number of cases varied among facilities, which may have introduced bias. This study reports survey results reflecting the policies and perspectives of individual institutions. To enhance its clinical significance, future research should define clinical evaluation criteria and collect detailed patient-level data for comprehensive analysis.

## 5. Conclusions

Since the approval of clazosentan in Japan, its use has increased, gradually replacing fasudil in clinical practice. While many institutions have reported a perceived benefit of clazosentan for preventing CVS, its impact on overall clinical outcomes remains inconclusive. Management strategies during clazosentan administration are becoming increasingly refined, and future studies are anticipated to contribute to the establishment of standardized protocols for the prevention of DCI following SAH.

## Figures and Tables

**Table 1:** Clinical characteristics of cases in each region of Japan.

	Total	Hokkaido	Tohoku	Kanto	Chubu	Kinki	Chugoku/Shikoku	Kyushu
Hospitals, No.	78	3	11	30	8	15	6	5
SAHs, No.	1316	21	186	670	144	161	83	51
Cliooing, No.	591	12	95	295	72	58	31	28
Coiling, No.	703	9	91	363	70	97	52	21
Clipping + Coiling, No.	22	0	0	12	2	6	0	2

## Data Availability

The datasets used and analyzed during the current study are available from the corresponding author upon reasonable request.

## Supplementary Materials

The following supporting information can be downloaded at: https://www.mdpi.com/article/10.3390/jcm14124107/s1, Supplementary Materials File: Survey Questions.

## Abbreviations

The following abbreviations are used in this manuscript:

CSF	Cerebrospinal fluid
CVP	Central venous pressure
CVS	Cerebral vasospasm
DCI	Delayed cerebral ischemia
EPA	Eicosapentaenoic acid
IRB	Institutional review board
IVC	Inferior vena cava
SAH	Subarachnoid hemorrhage
