# Supplementary material for: Japanese Nationwide Questionnaire Survey on the Treatment and Management of Subarachnoid Hemorrhage Due to Ruptured Cerebral Aneurysm
Source: J Clin Med. 2025 Jun 10;14(12):4107. doi: 10.3390/jcm14124107 (PMC12194453; doi:10.3390/jcm14124107)
Supplement: Supplementary file 1 [file jcm-14-04107-s001.zip › jcm-3645055-supplementary.pdf]

Request for Cooperation in a Survey on the Treatment and Management of Subarachnoid Hemorrhage due to Ruptured Cerebral Aneurysms

To Members of the Japan Society for Stroke Surgery

Dear Members,

We hope this letter finds you well.

With the cooperation of the Japan Society for Stroke Surgery, we are conducting a survey targeting facilities with certified technical instructors and specialists in order to assess the treatment and management of subarachnoid hemorrhage (SAH) due to ruptured cerebral aneurysms.

This survey will take approximately 15 minutes to complete, and we sincerely ask for your cooperation.

Survey Background and Objectives

As you may be aware, Clazosentan was approved for insurance-covered medical use in Japan in April 2022 as a preventive measure against cerebral vasospasm and is gradually being adopted in clinical practice. The 41st SAH/Spasm Symposium plans to include discussions on the use of Clazosentan.

This survey aims to elucidate the current practices regarding the prevention, treatment, and management of cerebral vasospasm following SAH since the introduction of Clazosentan and to compare the results with the findings from the 39th SAH/Spasm Symposium survey.

Survey Methodology

- No personal information will be collected in this survey.
- The survey will collect responses on institutional treatment policies and case numbers.
- In institutions with multiple certified technical instructors or specialists, a single representative is requested to respond.
- The survey will be conducted via Google Forms. Please use the URL or QR code below to complete the questionnaire.
- Responses are anonymous and cannot be withdrawn after submission.
- The deadline for responses is **October 31, 2024**.
- The survey is securely managed with SSL encryption and multiple layers of security.

Survey Link:

https://forms.gle/1QJzMQ65LZvwfMRQ8

Principal Investigator

Dr. Fusao Ikawa, Deputy Director, Neurosurgery, Shimane Prefectural Central Hospital

Chair, 41st SAH/Spasm Symposium

Collaborating Investigators

Dr. Motohiro Morioka, Professor, Department of Neurosurgery, Kurume University

Representative Organizer, SAH/Spasm Symposium

Dr. Mitsuto Mase, Professor, Department of Neurosurgery, Nagoya City University Hospital

Chair, 39th SAH/Spasm Symposium

Dr. Nobutaka Horie, Professor, Department of Neurosurgery, Hiroshima University

Vice Chair, 41st SAH/Spasm Symposium

Contact Information

Shimane Prefectural Central Hospital, Department of Neurosurgery

Address: 4-1-1 Himehara, Izumo, Shimane 693-8555, Japan

Tel: +81-853-22-5111

Fax: +81-853-21-2975

Email: fikawa-nsu@umin.ac.jp

Survey on the Treatment and Management of Subarachnoid Hemorrhage due to Ruptured Cerebral Aneurysms

This survey targets cases diagnosed with SAH due to ruptured cerebral aneurysms, where treatment was performed to prevent re-rupture within **72 hours of onset** between **January 1, 2023, and December 31, 2023**.

Consent for Research Use: Please check the box below if you agree to allow your responses to be used for research purposes.

☐ I agree that the responses in this survey may be used for research purposes.

Survey Questions

1. Institutional Information

Q1. Which regional branch of the Japan Neurosurgical Society does your institution belong to?

☐ Hokkaido

☐ Tohoku

☐ Kanto

☐ Chubu

☐ Kinki

☐ Chugoku-Shikoku

☐ Kyushu

2. Case Numbers

Q2-1. How many cases at your institution underwent rerupture prevention treatment within 72 hours of onset during the target period? ____ cases

Q2-2. Of the cases mentioned above, how many underwent craniotomy? ____ cases

Q2-3. How many underwent endovascular treatment? ____ cases

Q3. How many underwent a combination of craniotomy and endovascular treatment? ____ cases

3. Postoperative Management and Cerebral Vasospasm Prevention

Q4. Does your institution have a standardized protocol for postoperative management and vasospasm prevention?

☐ Yes (≥90%)

☐ Mostly standardized (75–90%)

☐ Partially standardized (50–75%)

☐ No, left to attending physicians.

4. Pharmacological Treatment

Q5. Which medications were administered to **≥80% of cases** within 14 days postcraniotomy?

☐ Fasudil

☐ Ozagrel

☐ Cilostazol

☐ Edaravone

☐ Statins

☐ Steroids

☐ Nicardipine (nonhypotensive use)

☐ Clazosentan

☐ EPA

☐ Others: ______

Q6. Which medications were administered to **≥80% of cases** within 14 days postendovascular treatment? (Same options as Q5)

Q7. Which medications were administered to **50–80% of cases** within 14 days postcraniotomy?

☐ Fasudil

☐ Ozagrel

☐ Cilostazol

☐ Edaravone

☐ Statins

☐ Steroids

☐ Nicardipine (nonhypotensive use)

☐ Clazosentan

☐ EPA

☐ Others: ______

Q8. Which medications were administered to **50–80% of cases** within 14 days postendovascular treatment? (Same options as Q5)

Q9. Are there any differences in the medications used for severe cases (WFNS grades IV and V) compared to mild cases (WFNS grades I–III)？

☐ Yes

☐ No

Q10. This question is for facilites that ansewerd "Yes" to question 9. Which medications were administered to **≥80% of cases** within 14 days postcraniotomy?

☐ Fasudil

☐ Ozagrel

☐ Cilostazol

☐ Edaravone

☐ Statins

☐ Steroids

☐ Nicardipine (nonhypotensive use)

☐ Clazosentan

☐ EPA

☐ Anesthetic drugs (ex. propofol)

☐ Others: ______

Q11. This question is for facilites that ansewerd "Yes" to question 9. Which medications were administered to **≥80% of cases** within 14 days postendovascular treatment? (Same options as Q10)

5. Cerebrospinal fluid drainage management

Q12. Does your institution use perfusion therapy* as a method of cerebrospinal fluid drainage?

*The cerebrospinal fluid drainage tube is used for purposes other than drainage, such as injecting medicine.

☐ Yes

☐ No

Q13. Which method of cerebrospinal fluid drainage is used postcraniotomy (please answer the method used in most cases)?

☐ Spinal drainage

☐ Cisternal drainage

☐ Spinal drainage ＋ cisternal drainage

☐ Spinal drainage ＋ ventricle drainage

☐ Cisternal drainage ＋ ventricle drainage

☐ Ventricle drainage

☐ No drainage

Q14. Which method of the cerebrospinal fluid drainage is used postendovascular treatment (please answer the method used in most cases)? (Same options as Q13)

6. Central Venous Pressure Measurement

Q15. Did you measure central venous pressure?

☐ Yes

☐ No

7. Clazosentan Usage

Q16. How many cases at your institution received clazosentan during the survey period? ____ cases

Q17. Is clazosentan used in combination with fasudil?

☐ Used together

☐ Not used together

☐ Initially used concomitantly, but concomitant use was discontinued

Q18. For facilities that have checked "Discontinued use," please select all the reasons.

☐ Respiratory complications, such as pulmonary edema

☐ Cardiac complications, such as heart failure

☐ Low blood pressure

Q19. How effective do you perceive clazosentan to be for preventing cerebral vasospasm?

☐ Highly effective

☐ Effective

☐ Uncertain

☐ Minimally effective

☐ Ineffective

Q20. How effective do you perceive clazosentan to be for improving outcomes? (Same options as Q19)

Q21. Of the cases in which clazosentan was administered, how many discontinued treatment?

____ cases

Q22. Select all the reasons why the administration of clazosentan was discontinued midway among the cases wherein it was administered.

☐ Respiratory complications, such as pulmonary edema

☐ Cardiac complications, such as heart failure

☐ Brain edema

☐ Other edema

☐ Others

Q23. Which trend describes cases wherein clazosentan administration was discontinued.

☐ Gradually decreasing

☐ Gradually increasing

☐ No change

Q24. For facilities that checked "Gradually decreasing" in question 23, select all the reasons.

☐ Improved water balance management

☐ Weight measurement started

☐ Not administered if cardiac function is impaired

☐ Not administered in severe cases

☐ Not administered to the elderly

Q25. How many cases have you experienced wherein cerebral vasospasm of ≥50% stenosis required treatment despite the administration of clazosentan?

____ cases

Q26. What percentage of cases with cerebral vasospasm of ≥50% stenosis required treatment despite the administration of clazosentan?

☐ <0–10%

☐ <10–30%

☐ <30–50%

☐ ≥50%

Q27. Select all of the factors that explain cases wherein cerebral vasospasm of ≥50% stenosis requiring treatment occurred despite the administration of clazosentan.

☐ Age <50 years

☐ Male sex

☐ Female sex

☐ History of diabetes

☐ Age ≥75 years

☐ Craniotomy

☐ Endovascular treatment

Q28. Select the future policy on clazosentan use.

☐ In principle, use in all cases

☐ Select the cases to use

☐ No idea

☐ In principle, do not use

Final Statement

Thank you for your cooperation. The results of this survey will be discussed at the

SAH/Spasm Symposium at Stroke 2025.
